# Rabbit aortic aneurysm model with enlarging diameter capable of better mimicking human aortic aneurysm disease

**DOI:** 10.1371/journal.pone.0198818

**Published:** 2018-06-11

**Authors:** Yonghua Bi, Hongmei Chen, Yahua Li, Zepeng Yu, Xinwei Han, Jianzhuang Ren

**Affiliations:** 1 Department of Interventional Radiology, the First Affiliated Hospital of Zhengzhou University, Zhengzhou, China; 2 Department of Histology&Embryology, Medical College of Zhengzhou University; Department of Ultrasound, Zhengzhou Central Hospital Affiliated to Zhengzhou University, Zhengzhou, China; Max Delbruck Centrum fur Molekulare Medizin Berlin Buch, GERMANY

## Abstract

The self-healing phenomenon can be found in the elastase-induced abdominal aortic aneurysm (AAA) model, and an enlarging AAA model was successfully induced by coarctation. Unfortunately, aortic coarctation in these enlarging models is generally not found in human AAA disease. This study aimed to create an experiment model of enlarging AAA in rabbits to better mimic human aortic aneurysm disease. Eighty-four male New Zealand white rabbits were randomly divided into three equal groups: two aneurysm groups (A and B) and a SHAM group. Aneurysm group rabbits underwent extrinsic aortic stenosis below the right renal artery and received a 10-minute incubation of 60 μl elastase (1 unit/μl). Absorbable suture was used in Group A and nonabsorbable cotton thread was used in Group B. A sham operation was performed in the SHAM group. Aortic diameter was measured after 1, 3, 7, and 15 weeks; thereafter animals were sacrificed for histopathological, immunohistochemical and quantitative studies. Two rabbits died at 29 and 48 days, respectively, after operation in Group B. All aneurysms formed and enlarged progressively by 3 weeks in the Aneurysm groups. However, diameter enlargement in Group A was significantly lower than that in Group B at 7 weeks. Aneurysm groups developed intimal hyperplasia; intima-media thickness (IMT) increased significantly by week 7, and aortic media thickness and intima-media ratio (IMR) increased significantly by week 15. Marked destruction of elastin fibers and smooth muscle cells (SMCs) occurred 1 week later and increased progressively thereafter. Intimal hyperplasia and SMCs content in Group A increased significantly by week 15 compared with Group B. Aneurysm groups exhibited strong expression of matrix metalloproteinases 2 and 9 and RAM11 by week 1, and decreased progressively thereafter. In conclusion, this novel rabbit AAA model enlarges progressively without coarctation and is capable of better mimicking human aortic aneurysm disease.

## Introduction

Abdominal aortic aneurysm (AAA), a life-threatening disease, is characterized by chronic inflammation and remodeling of aortic wall tissue [1]. However, the pathogenesis of AAA remains poorly understood [2]. Experimental AAA models have been developed in small animals to mimic human AAA disease. The small animal models have been classified into three categories: chemical injury aneurysm model, genetically predisposed animal model, and hemodynamically-induced aneurysm model [3]. Chemical injury aneurysms induced by elastase and calcium chloride are popular models [4–13]. However, elastase and calcium chloride does not always induce AAA formation [14–16]. It has been reported that aneurysm diameter increases no more than 70% [5,7,8] and even heals spontaneously in elastase-induced AAA models [7]. Human AAA will enlarge progressively and cause aneurysm rupture or even death [17]. A new kind of enlarging AAA model was successfully induced in rats [18] and rabbits [19] to successfully overcome the self-healing phenomenon reported by Origuchi et al. [7]. Unfortunately, aortic coarctation in these enlarging models is generally not found in human AAA disease, which also poses difficulty for further interventional procedures, such as stent implantation. In this study, we modified this method and induced rabbit AAA model with enlarging diameter to better mimic human aortic aneurysm disease.

## Materials and methods

### Reagents and materials

Porcine pancreatic elastase (1 unit/μl, ≥30 units/mg, PI 9.5, PH 8.1–8.9), was from Shanghai Kayon Biological Technology Co., Ltd (Shanghai, China). Elastic van-Gieson (EVG) was from Baso (Zhuhai, China). Picrosirium red (PSR) and Mouse monoclonal anti-alpha-smooth muscle actin [1A4] (A2547) were from Sigma-Aldrich (Shanghai) Trading Co.,Ltd (Shanghai, China). Monoclonal Mouse Anti-Rabbit Macrophage (Clone RAM11, Code M0633) and Monoclonal Mouse Anti-Human CD31 (Clone JC70A, Code IR610) were from Dako (Denmark). RAM11 was used to recognize macrophages infiltration in connective tissue and aortic wall. Anti-MMP2 antibody [4D3] (ab2462) and Anti-MMP9 antibody [56-2A4] (ab58803) were from Abcam (Hong Kong, China); The UltraSensitive streptavidin-peroxidase kit (KIT-9701) and diaminobenzidine tetrahydrochloride (DAB) were from Maixin Bio. (Fuzhou, China). The absorbable surgical polyglycolic acid (PGA) suture (Trade name PGA Jinjie) was purchased from Shanghai Pudong gold ring medical supplies limited by Share Ltd., Shanghai, China. This PGA suture is coated with poly (ethylene glycol) lactone and octadecoic acid without any pro-inflammatory material.

### Animals and experimental groups

Eighty-four male New Zealand white rabbits were equally and randomly divided into three equal groups: Group A, Group B and a SHAM group. Rabbits in Aneurysm groups A and B underwent an extrinsic stenosis below the right renal artery and received a 10-minute incubation of 60 μl elastase (1 unit/μl). Absorbable suture was used in Group A, which was absorbed 4 weeks later to terminate the aortic coarctation; balloon dilation was performed 8 weeks later if stenosis still persisted. Nonabsorbable cotton thread was used in Group B. A sham operation was performed in the SHAM group. All experimental animals had access to water and rabbit chow *ad libitum*. This study was conducted under the approval of the Animal Experimental Ethics Committee of Zhengzhou University (permit number: keyan-2015-002). All surgery was performed under sodium pentobarbital anesthesia and all efforts were made to minimize suffering.

### A novel AAA model

A midline laparotomy was performed under sterile conditions after intravenous injection of 30 mg/kg sodium pentobarbital for anesthesia. A 1.5-cm segment of infrarenal abdominal aorta that contained fewer branches was carefully dissociated and wrapped with a piece of sterile gauze. The abluminal surface of this segment was then incubated with porcine pancreatic elastase (60 μl, 1 unit/μl) for 10 minutes in Aneurysm groups. Then the gauze was removed and the incubated segment was irrigated twice with physiological saline. A ligature was performed just above the incubated segment with similar stenosis of 1.3 mm in diameter in the Aneurysm groups. Segment was incubated with 60 μl of physiological saline for 10 minutes and a ligature was performed but removed immediately in SHAM group. All rabbits were systemically heparinized with 200 IU/kg low-molecular-weight heparin sodium (Shanghai No.1 Biochemical Pharmacology Co. Ltd, Shanghai, China) during experimental period.

### Intravenous digital subtraction angiography

Rabbits underwent intravenous digital subtraction angiography (IVDSA) imaging after 1, 3, 7, and 15 weeks similar to previous reports [9,12,13,20]. Specifically, a 22-G angiocatheter was placed in the ear vein for 5s DSA scanning (Siemens Artis Zee, Germany). Iodinated contrast material (9 ml) was injected into the ear vein to visualize the AAA. 3D-DSA analysis and diameter measurement were performed with RadiAnti DICOM Viewer 3.4.2 (Medixant Company, Poznan, Poland), which is an application for processing and displaying medical images in DICOM (Digital Imaging and Communications in Medicine) format. Successful AAA formation was defined increase of at least 50 percent in the focal dilation ratio of the incubated aorta compared to its normal diameter.

### Animal sacrifice and histopathology

Every 7 rabbits in each group were sacrificed for histopathology after IVDSA at 1, 3, 7, and 15 weeks, except for two rabbits in Group B that died at 29 and 48 days, respectively. Animals were anesthetized again, and pressure perfusion fixed with 4% buffered paraformaldehyde was processed. Aortic tissues were embedded in paraffin and cut into 5-μm sections. To examine the overall morphology and the distribution of elastic lamellae, hematoxylin-eosin and elastic van-Gieson (EVG) staining was performed. Paraffin sections were stained according to the manufacturer’s instructions.

### Smooth muscle density with immunofluorescence detection

After blocking endogenous peroxidases, paraffin sections were processed and incubated at 4°C overnight with mouse monoclonal anti-alpha-smooth muscle actin (1:150 diluted in PBS). Aortic sections were subsequently incubated with fluorescein isothiocyanate-conjugated goat antimouse IgG and to detect the assembly of smooth muscle cells (SMCs).

### Immunohistochemical analysis

Tissue sections were deparaffinized in xylene and rehydrated through graded alcohol washes. Sections were incubated with 1% H_2_O_2_ in methanol and 10% goat serum for 30 min to block endogenous peroxidase activity and non-specific binding. The primary antibodies to MMP2 (1:200 dilution), MMP9 (1:300 dilution), and RAM11 (1:100 dilution) were incubated overnight at 4°C. According to the manufacture’s protocol, sections were then incubated with biotinylated anti-mouse second antibody for 20 min followed by the SP method. Sections were visualized with diaminobenzidine tetrahydrochloride and counterstained with hematoxylin.

### Western blot for MMP2, MMP9 and RAM11

Aortic tissues were lysed with RIPA protein lysis buffer (Code P0013B, Beyotime Biotechnology, Shanghai, China). Total protein was separated on 10% SDS-PAGE and then transferred to a polyvinylidene difluoride membrane of 0.45μm (Code IPVH00010, Millipore Corporation, Billerica, MA., USA). The membrane was blocked with 5% milk solution and then probed with primary antibodies (MMP2, MMP9 and RAM11) overnight at 48°C. The membrane was incubated with an HRP-conjugated secondary antibody (dilution 1:50000) for 2 h at 37 °C after being washed 5–6 times. An enhanced chemiluminescence kit (Applygen Technologies Inc., Beijing, China) was used to visualize the protein band. The β-actin was used as an internal control for all bots.

### Quantification analyses

Aortic lumen perimeter, area of medial SMCs and elastin were measured by Image Pro-Plus 6.0 software (Media Cybernetics, Rockville, MD) as previously described [21]. At least 1 slide of good staining per rabbit and 6 non-overlapping fields per slide was analyzed under ×400 magnification. The media thickness, intima thickness and intima-media thickness (IMT) were measured as the average thickness of 6 fields of cross-sections. Semiquantitative analyses for elastin and SMCs content were calculated as the mean area of 6 fields of a cross section. Densitometric analysis of lytic bands was performed by public domain software NIH Image version 1.61.

### Statistical analysis

All data were expressed as means ± SD. One-way ANOVA and two-way ANOVA followed by Bonferroni post-hoc tests were used for statistical analysis (Prism 5.0, GraphPad Software, Inc., SanDiego, CA., USA). Differences were considered statistically significant when *p* < 0.05.

## Results

### Inner diameter follow-up by IVDSA

**Fig 1:**
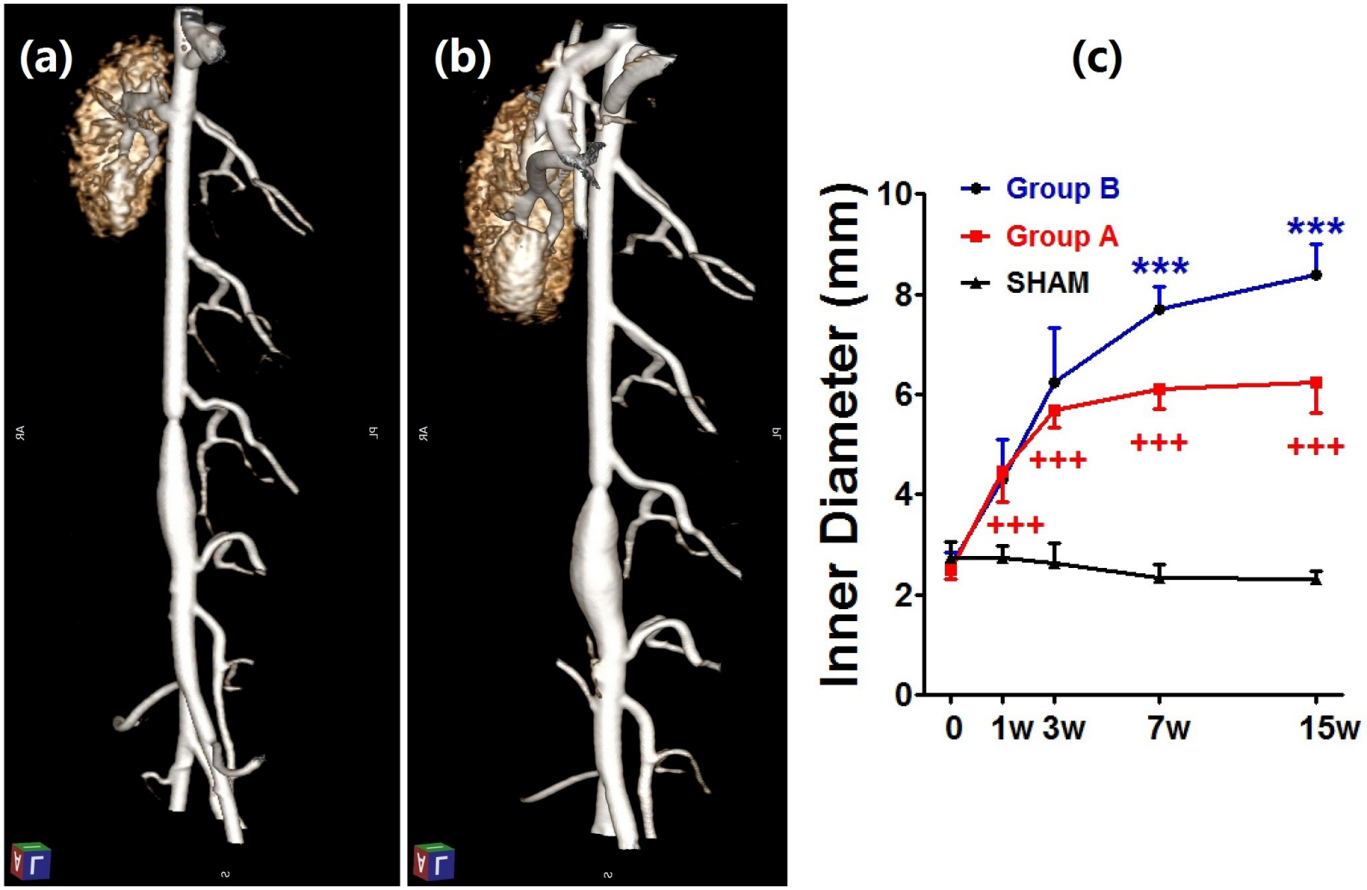
Follow-up of aortic diameter by IVDSA. Aneurysm and proximal stenosis was obvious in Group A after 3 weeks (a). Aneurysm enlarged further and stenosis disappeared in Group A after 15 weeks (b). (c) Profile of diameter changes indicated that Aneurysm groups dilated progressively, but diameter enlargement in Group A was significantly lower than in Group B at 7 weeks. *** *p* < 0.0001, Group B compared with Group A and SHAM group; +++ *p* < 0.0001, Group A compared with SHAM group.

All rabbits survived the surgical procedure. Two rabbits in Group B died 29 and 48 days, respectively, after operation. Compared with the SHAM group, aneurysm diameters in the Aneurysm groups increased significantly after 1 week (*p* < 0.0001). In Group A, the suture was absorbed in 13 rabbits about 4 weeks later; the rest of 3 rabbits showed remained stenosis after 8 weeks, and balloon dilation was performed to terminate the aortic coarctation. Interestingly, AAA still enlarged gradually in Group A after 4 weeks. All aneurysms formed by 3 weeks in Group A (5.7 ± 0.3 mm) and Group B (6.2 ± 1.1 mm), aneurysm enlarged progressively, and the diameters increased to 6.2 ± 0.6 mm in Group A and 8.4 ± 0.6 mm in Group B at 15 weeks, respectively. However, diameter enlargement in Group A (6.1 ± 0.4 mm) was significantly lower than that in Group B (7.7 ± 0.4 mm) at 7 weeks. No aneurysm was seen in the SHAM group, diameter remained stable during follow-up. (Fig 1)

### Changes in aortic wall thickness

**Fig 2:**
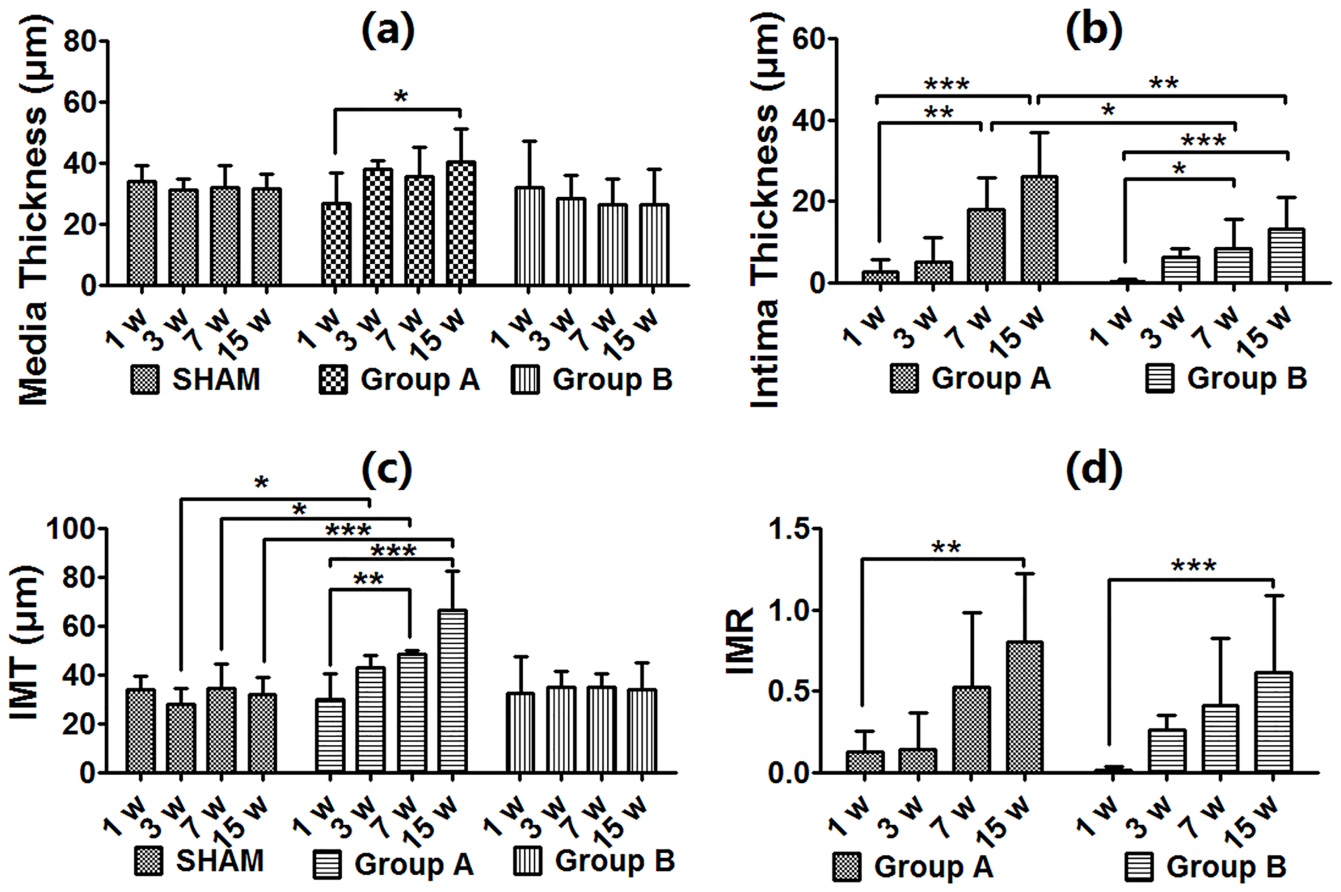
Profiles of aortic lumen perimeters. Media thickness increased significantly by week 15 in Group A (a); Intimal hyperplasia increased significantly by week 7 in the Aneurysm groups. Group A increased significantly by week 15 compared with Group B. (b); IMT increased significantly by week 7 in Group A (c); IMR increased significantly by week 15 in the Aneurysm groups (d). * *p* < 0.05, ** *p* < 0.01, *** *p* < 0.0001. IMR = intima-media ratio; IMT = intima-media thickness.

Media thickness of aortic wall in Group A thinned by 1 week (27.0 ± 10.0 μm), but thickened significantly 15 weeks later (40.6 ± 4.1 μm, *p* < 0.05), although this change was not significant compared with the SHAM group or Group B. Media thickness thinned gradually in Group B, although the decrease was not significantly (Fig 2a). Intimal hyperplasia occurred in the Aneurysm groups. Intimal thickness by weeks 7 and 15 increased significantly compared with week 1 (*p* < 0.05). Interestingly, the degree of intimal hyperplasia in Group A by weeks 7 and 15 was significantly higher compared with in Group B (*p* < 0.05, Fig 2b). Overall thickness of the aortic wall in Group A, calculated by intima-media thickness (IMT), increased significantly by week 7 (versus week 1, *p* < 0.01) and by week 3 it was higher than the SHAM group (*p* < 0.05). Group B and the SHAM group showed stable IMT. (Fig 2c) Intimal hyperplasia was also calculated by intima-media ratio (IMR), and showed similar results. IMR increased gradually and was showed higher by week 15 than in week 1 in the Aneurysm groups (*p* < 0.01). The SHAM showed no intimal hyperplasia. (Fig 2d and S1 Table)

### Changes in elastic lamellae

**Fig 3:**
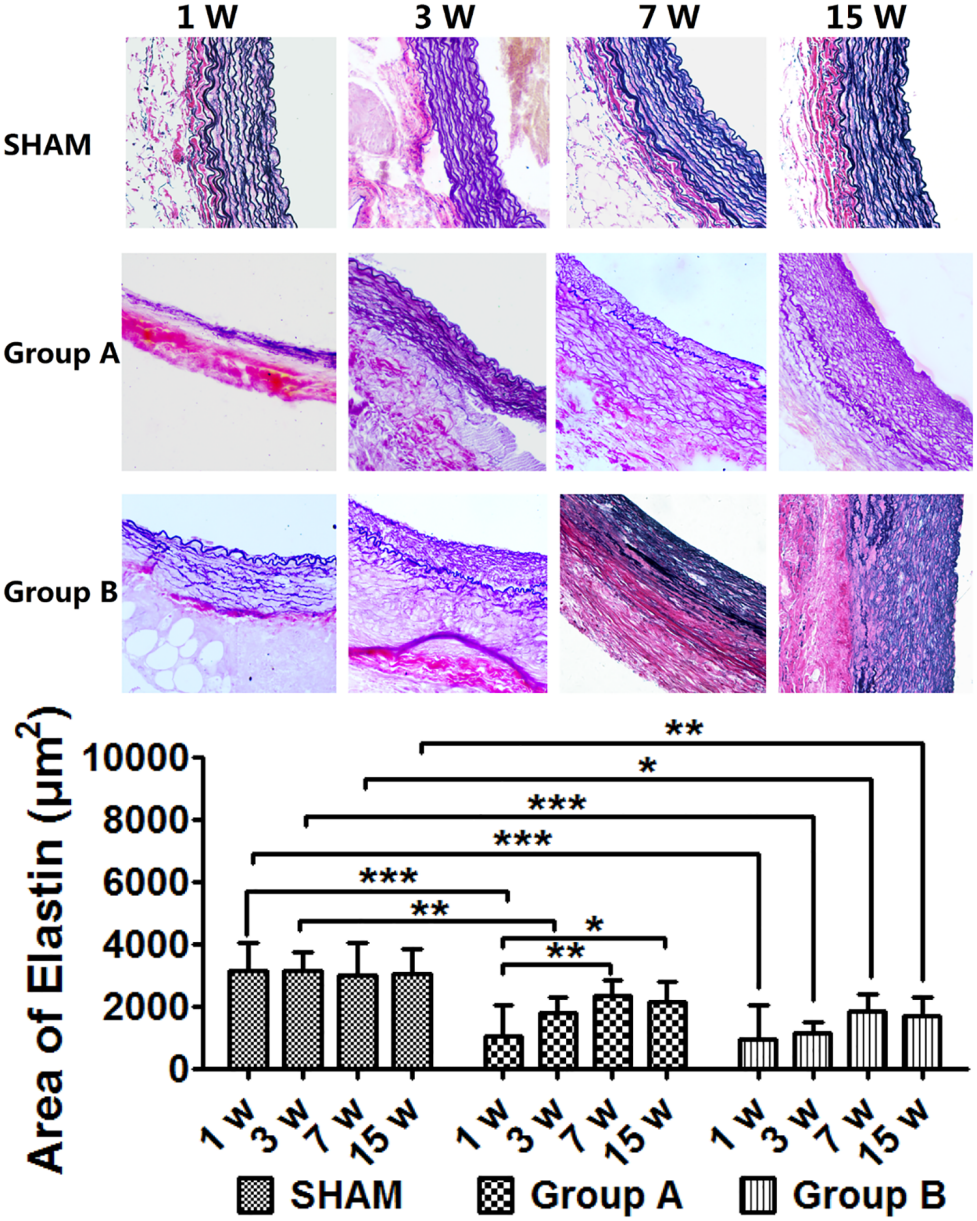
Profiles of elatin content change by EVG staining. Elastin fibers were destroyed markedly by week 1, and elastin increased progressively thereafter. **p < 0.01, ***p < 0.0001. Original magnification ×400.

Elastic van-Gieson staining for elastic fibers showed marked destruction in Group A. Elastin contents were significantly lower than those in the SHAM group by week 1 (*p* < 0.0001) and by week 3 (*p* < 0.01). However, compared with week 1, elastin content showed a tendency to increase, which increased significantly by week 7 (*p* < 0.01). Elastic fibers were also destroyed significantly in Group B; however, elastin content remained stable during follow-up, although it was lower compared with the SHAM group (*p* < 0.05). Elastin content did not change significantly in SHAM group during follow up. (Fig 3 and S2 Table)

### Changes in vascular SMCs content

**Fig 4:**
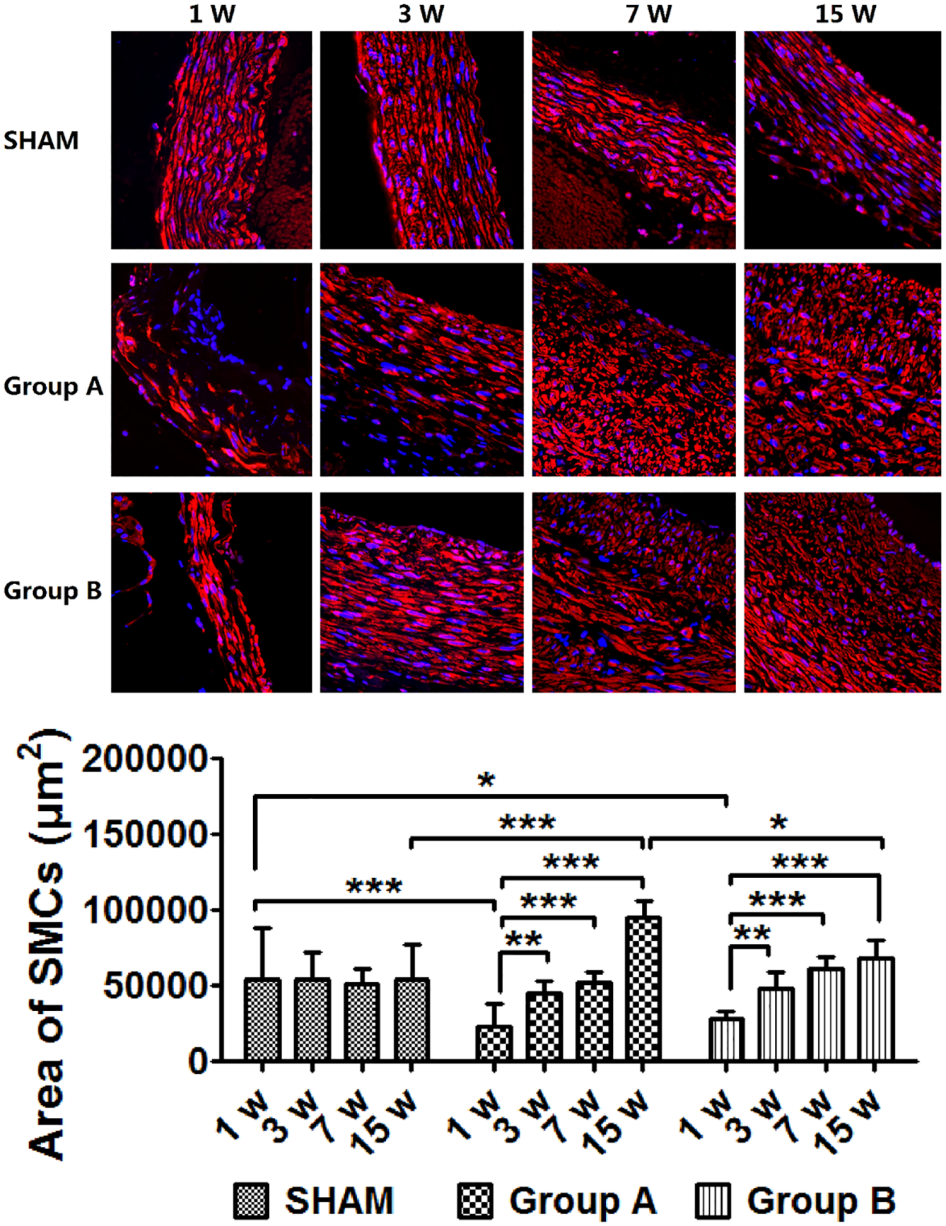
Smooth muscle cells changes and the profiles of content change. SMCs were destroyed markedly after 1 week, but increased progressively thereafter. SMCs content in Group A increased significantly by week 15 compared with Group B. **p < 0.01, ***p < 0.0001. Original magnification ×400.

Vascular SMCs content, calculated by the area of SMCs per high-power field, decreased significantly after 1 week in Group A (*p* < 0.0001) and Group B (*p* < 0.05) compared with the SHAM group. However, both groups increased significantly by week 3 (versus week 1, *p* < 0.01). SMCs content showed a similar tendency to increase in the Aneurysm groups. Compared with the control group, vascular SMCs in Group A and Group B increased significantly by week 15 (*p* < 0.0001 and *p* < 0.05, respectively). (Fig 4 and S3 Table)

### MMP2, MMP9 expression and inflammatory infiltration

**Fig 5:**
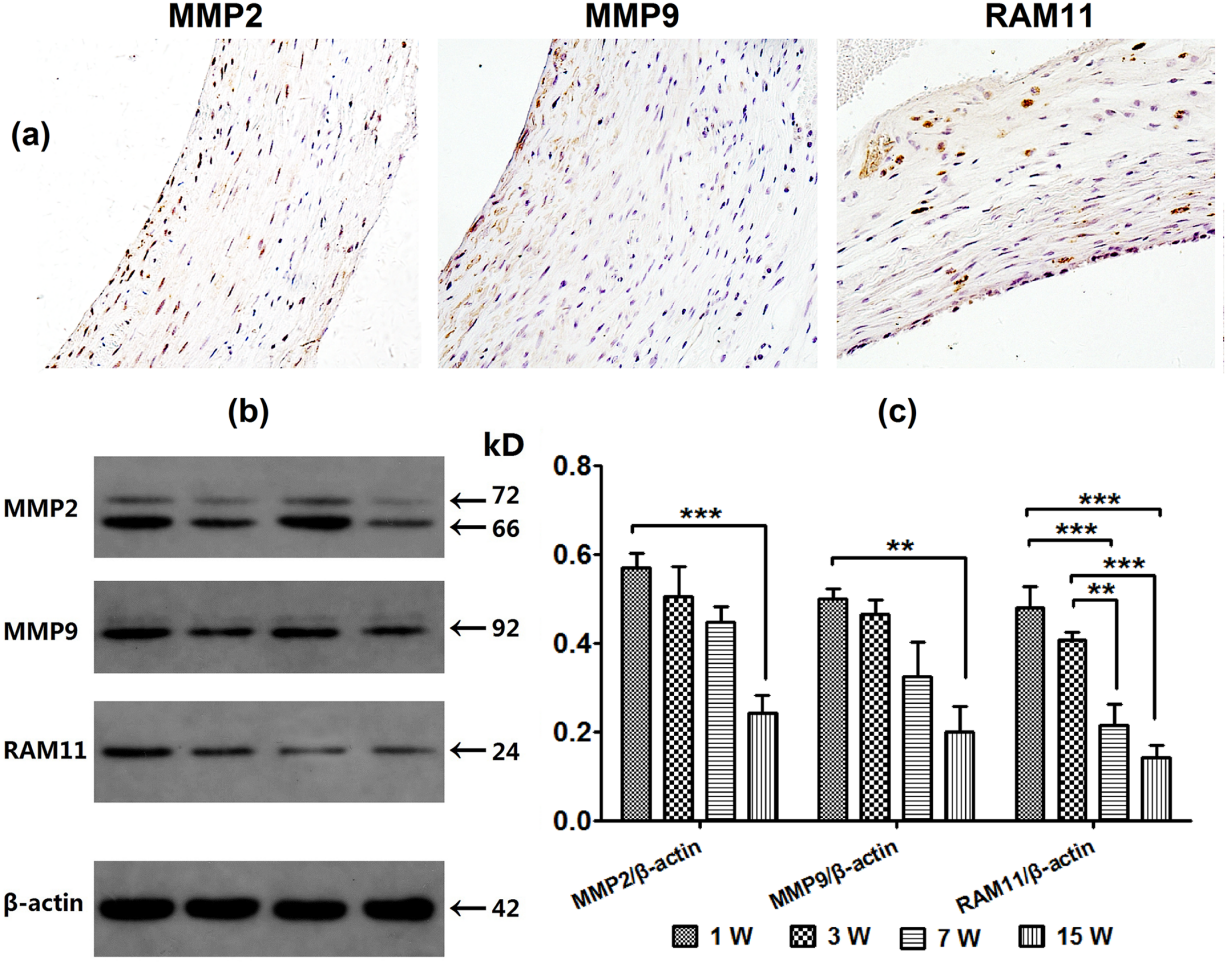
Immunohistochemical staining and quantitative analysis of MMP2, MMP9, and RAM11 for Group A. (a) Immunohistochemical staining. (b) Western blot test. (c) All expression increased by week 1, and decreased significantly by week 15.* *p* < 0.05, **p < 0.01, ***p < 0.0001.

Both aneurysm groups showed increased MMP2 activity relative to the SHAM group by week 1 and week 3, and maintained moderate expression thereafter. MMP9 expression was higher in the Aneurysm groups compared to the SHAM group by week 1, and expression decreased after 3 weeks, resulting in no difference with the SHAM group. Infiltration of a few macrophages was detected by RAM11 in the Aneurysm groups, and was mainly confined within the adventitia. This was higher relative to the SHAM group by week 1, and decreased to weak expression after 7 weeks. Quantitative analysis of MMP2, MMP9, and RAM11 for Group A are shown in Fig 5. All expression increased by week 1, and decreased significantly by week 15 (S4 Table).

## Discussion

All aneurysms formed 3 weeks after a combination of elastase incubation and absorbable suture caused stenosis. The suture absorbed about 4 weeks later to terminate the aortic coarctation, and rabbit AAA model remained stable thereafter. The diameter increased to 6.2 ± 0.6 mm by the end of follow up (15 weeks) and was significant higher than that of the elastase-induced model. This novel AAA model showed typical hisopathological/immunohistochemical changes, MMP expression, and inflammatory infiltration. Elastin fibers and SMCs were destroyed markedly, with strong expression of matrix metalloproteinase 2, 9 and RAM11 1 week later. However, intimal hyperplasia, elastin fibers and SMCs content increased progressively thereafter. These complex changes are quite helpful for further investigation of the pathogenesis of AAA formation and progression.

In a previous experiment, we found that aneurysm did not form after low concentration elastase incubation (0.1–5 units/μl) for half an hour, [9] unless aortic wall was destroyed dramatically by high concentration of elastase [19]. This fast-induced model enlarged more than 50% within 1 week, but the aneurysm was unstable and soon returned to normal condition [14]. Origuchi et al. [7] first reported this self-healing phenomenon in the traditional elastase-induced AAA model, but our model successfully overcame this phenomenon. Roach et al. [22] reported that arterial dilation is reversible upon removal of the coarctation for poststenotic dilatation in the femoral arteries of dogs, but this is not consistent with our findings. In our study, although the rate of increase was lower than in Group B, AAA diameter still enlarged progressively after stenosis disappeared 4 weeks later in Group A. This indicates that turbulence flow caused by coarctation may be essential for aneurysm initiation, and may upregulate MMP2 and MMP9 expression to degrade cell basement and internal elastic lamina [23]. This stable model can provide a novel model for the study of the formation and development of human AAA.

A new kind of enlarging AAA model was successfully induced in rats [18] and rabbits [19] to successfully overcome the self-healing phenomenon. Besides, Molácek et al. reported that increased turbulence flow caused by the stenosing cuff after infusion of elastase induced AAA in the pig [24]. Unfortunately, aortic coarctation in these enlarging models generally is not found in human AAA disease, and this creates difficulty for further interventional procedures, such as stent implantation. For example, partially covered polyester stent grafts were successfully implanted in rabbit aortas [25]. In this study, we modified this method by using an absorbable suture and successfully induced stable rabbit AAA model without coarctation. This model can be used to test aneurysm repair by covered stent grafts in our future studies. Besides, elastase incubation within aorta rather than outside aorta has been widely used in experimental AAA creation [4]. We altered the elastaste administration (periaortic incubation instead of intraluminal perfusion) and obtained some advantages over an intraluminal elastase perfusion-induced aneurysm. Intraluminal elastase perfusion requires the use of a cannula in the common iliac artery and should avoid leakage of the elastase solution during perfusion. The improved method showed the advantages of simple operation, short modeling time, little trauma and high survival rate.

Some limitations need to be addressed. First, echography to assess vortexing changes was not performed in this study. Post-stenosis vortexing persisted in Group B, but progressively decreased with the resorption of the suture material at 3 weeks in Group A. Second, this model did not develop an intraluminal thrombus and increase in SMC and medial thickness were found, which were different from human AAA. Third, we did not evaluate atherosclerosis, and it would be beneficial to test the effect of cholesterol intoxication on this model. AAA processes relevant to the human situation develop up to about 3 week, and thereafter processes that may be termed "regenerative" develop and should be addressed in the chronological use of this model. The regenerative processes initiated early may be related to the healing process in this model. The long term outcome of our model still needs to be confirmed, although it was stable by week 15 in this study. This novel AAA can dilate progressively and permits further interventional procedures, which may better mimic human aortic aneurysm disease.

## Supporting information

S1 Table Change of aortic wall thickness and statistical analysis results. (XLSX)

S2 Table Changes in elastic lamellae and statistical analysis results. (XLSX)

S3 Table Changes in vascular SMCs content and statistical analysis results. (XLSX)

S4 Table Western blot test and statistical analysis results. (XLSX)
